# Survey data on public perceptions of salmon aquaculture industry in Norway, Tasmania, and Iceland

**DOI:** 10.1016/j.dib.2024.110067

**Published:** 2024-01-15

**Authors:** Marit Schei Olsen, Eirik Mikkelsen, Karen A. Alexander, Ragnheidur Thorarinsdottir, Tonje C. Osmundsen

**Affiliations:** a NTNU Social Research, Trondheim, Norway; b Nofima, Tromsø, Norway; c Centre for Marine Socioecology, University of Tasmania, Hobart, Tasmania 7001, Australia; d Institute for Marine and Antarctic Studies, University of Tasmania, Hobart, Tasmania 7001, Australia; e International Centre for Island Technology, Heriot Watt University, Back Road, Stromness, Orkney KW16 3AW, UK; f Rector's Office, Agricultural University of Iceland, 311 Hvanneyri, Iceland; g Faculty of Civil and Environmental Engineering, University of Iceland, 102 Reykjavik, Iceland

**Keywords:** Social license to operate, Social acceptance, Public opinion, Fish farming, Mariculture, Questionnaire

## Abstract

This article presents data collected using online surveys conducted in Norway, Tasmania (Australia) and Iceland, with the aim of exploring public perceptions of the salmon aquaculture industry in each country. A total of 2085 survey participants provided responses, with 1183 from Norway, 406 from Tasmania, and 496 from Iceland.

The survey encompassed various aspects of attitudes towards and perceptions of the aquaculture industry. Participants were asked questions regarding their environmental concerns, trust in governance and management, and knowledge of the aquaculture industry in their respective country. Additionally, attitudes towards the industry were explored using questions related to preferences regarding information sources, perceptions of industry contributions, distribution of economic benefits, financial significance in local community, sustainability, and acceptance and tolerance of industry production. Respondents were also given the opportunity to provided text comments regarding the areas in which they thought the industry should become more sustainable. Demographic data on the respondents were collected, directly from the participants and from existing panel data from the survey company. However, the dataset excludes information on residence on the local level (postal code) to ensure anonymity of the respondents.

The survey design was created by the SoLic-project (2019-2022, supported by the Research Council of Norway, no. 295114), drawing on the social license literature and the team's extensive research experience on topics related to the aquaculture industry, social acceptance, and legitimacy.

The dataset presented in the article combines raw survey data with additional analysis data derived from grouping answer options or recoding data variables. The data provided in this article offers a valuable resource for researchers, industry representatives, public authorities, and other parties interested in salmon aquaculture. It enables comparative analyses and further investigations into public perceptions in Norway, Tasmania, and Iceland. This dataset can be used to explore a wide range of topics and extend the research conducted by the SoLic project team.

Specifications Table

**Table:** 

Subject	*Social sciences*
Specific subject area	*Attitudes towards salmon aquaculture and perceptions of its impacts and governance*
Data format	Raw. Additional simplified variables created by consolidating raw data.
Type of data	Table (.csv format) Supporting materials (codebook and text version of survey)
Data collection	Data were collected by survey companies in Norway, Iceland and Tasmania (Australia), which recruited respondents by distributing e-mail invitations to their panels. The research group established minimum respondent quotas for each region, with individuals under the age of 18 being excluded from participating in the survey.
Data source location	Country: Norway, Australia (Tasmania), Iceland.
Data accessibility	Repository name: Zenodo Data identification number: https://doi.org/10.5281/zenodo.10302174 Direct URL to data: https://zenodo.org/records/10302174
Related research article	Olsen, M.S., Amundsen, V.S. and Osmundsen, T.C. (2023). Exploring public perceptions and expectations of the salmon aquaculture industry in Norway: A social license to operate? Aquaculture, 574, 739632. https://doi.org/10.1016/j.aquaculture.2023.739632

## 1 Value of the Data

• The data offers valuable insights into public perceptions and attitudes towards the salmon aquaculture industry in the respective countries, specifically regarding the concepts of social license to operate, social acceptance, and legitimacy. The data provides cross-country comparisons that can serve to validate or challenge the existing body of research concerning concepts of social acceptability and social license to operate for aquaculture industry.
• The data provides extensive sample sizes for examining local perceptions in selected counties in Norway, as well as statistically significant sample sizes for national perceptions in Norway, Tasmania (Australia), and Iceland, and allows for comparative analysis of public attitudes across these three salmon-producing countries. By employing a consistent set of questions across all three countries and conducting surveys simultaneously, the data offers insights into public opinion and discourse, highlighting contextual variations. The data facilitates the identification of trends and patterns that might be overlooked when studying a single cultural context. Consequently, it leads to more generalized and robust findings, enabling a broader, more global perspective.
• Researchers and educators can utilize the data to conduct further analysis of perceptions, replicate existing studies, and enhance their analyses though additional investigations. While some published and forthcoming articles have utilized the data, these analyses were not exhaustive. Various methodological approaches can be employed to analyze this dataset.
• The data serves as a fundamental resource for researchers and decision-makers interested in studying public attitudes towards aquaculture industry. It can be supplemented with additional data, such as information related to industry production, value creation, demographic variables, and more.

## 2 Background

The survey aimed to explore the determinants and mechanisms behind the level of social license to operate for the aquaculture industry. Drawing upon relevant academic literature on social license, as described in Olsen et al. [1], our objective was to gather data on attitudes towards the industry and expand our understanding of the factors influencing its social license to operate. We also adapted previous survey designs measuring public attitudes to suit the context of aquaculture in the regions data were collected for.

The data collected pertains to both macro and micro levels, encompassing perceptions of the industry at both the national and local level community levels. Key concepts covered in the survey include transparency, visibility, and credibility, as well as the industry's contributions and benefits to local communities and the broader state level. Furthermore, it examines the perceptions of the industry's willingness to meet the expectations and interests of authorities and society.

In addition to the published articles by Olsen et al. [1] and Misund et al. [2], this dataset offers more detailed insights into perceptions of the aquaculture industry and enables further investigations and analysis of the Norwegian data, as well as facilitating comparative analyses with other regions.

## 3 Data Description

The files associated with this data-in-brief article includes:

(1) Overview of survey questions and answer options (Survey.doc): The survey encompassed 28 questions related to the aquaculture industry, along with demographics, respondents’ knowledge of industry, trust in governance system, and environmental concerns. Some demographic variables were sourced from the existing panel data, while others were provided to respondents for their input.
(2) The raw survey data in .csv file format (Dataset Solic_2085 respondents.csv): The survey data file contains 71 variables and data from each of the 2085 respondents. Blank entries in the dataset indicate either a lack of response from the respondents or that specific questions were not applicable to certain respondents (questions exclusively posed to respondents in one country).
(3) The codebook in Word format (Codebook.doc): The codebook provides explanations and details regarding all variables included in the survey data file. It includes coding information for each survey question, response options provided in the raw data, and further clarifies the purpose and origin of variables computed by the research group (e.g., variable on aquaculture municipality) or the survey company (e.g., weight variables for data from Norway and Iceland). When used in conjunction with the raw data, this codebook serves as a valuable guide for navigating the dataset.

The dataset comprises responses from a total of 2085 respondents, with 1183 participants from Norway, 406 from Tasmania (Australia), and the remaining 496 from Iceland. All respondents were presented with questions in their respective native language, namely Norwegian, English and Icelandic.

The survey questions covered these main themes:

- Individual characteristics of respondents
- General confidence in the country's governance system, respondents’ concern for environmental issues, and self-assessed level of knowledge of the salmon aquaculture industry
- Availability of information about the industry, and the respondents’ most important information sources about the industry
- Perceptions about the salmon aquaculture industry (transparent, trustworthy, general impression, national importance, types of contributions)
- Perceptions about the distribution of benefits from the industry
- Perceptions about the environmental and other sustainability impacts of the industry
- Perceptions of the industry's behavior compared to expectations of society and authorities
- Confidence in how the authorities regulate the industry
- Level of social acceptance/social license for the industry
- The industry's relation to the local region where the respondent lives (financial contribution and contribution to local development)
- Contact with and impression of the industry locally

Questions concerning the aquaculture industry (Q1-Q20 in Survey.doc) was provided to all respondents. Additionally, respondents from two counties (*Troms* and *Hordaland*) in Norway, as well as all respondents from Tasmania and Iceland were asked questions concerning their contact with local aquaculture company (Q21-Q28).

There were some variations in the available response options for “I don't know/I don't want to answer/uncertain” for the three regions. Specifically, Norwegian respondents had the option of selecting “I don't know”, while Icelandic participants could choose between “I don't know” and “I don't want to answer”, or even opt to provide no response. However, respondents from Tasmania were not offered the “I don't know” option for all questions. During the analysis process, all instances of “I don't know” options are treated as missing data.

The demographic characteristics of the respondents, including age, gender, education, and area of residency, are displayed in Table 1 (Norway), Table 2 (Tasmania), and Table 3 (Iceland). These tables showcase the variations in demographics stemming from differences in options, such as the educational descriptions, as well as variances in the number of demographic variables provided by the survey company in each country (not all variables are listed in the tables below)^1^.

**Table 1:** Overview of respondent distribution in terms of age, gender, area of residency, county, and education for Norway data.

Variable		N	Percent
Age group (years)			
	18-20	23	1,9
	21-24	49	4,1
	25-29	80	6,8
	30-34	67	5,7
	35-39	140	11,8
	40-44	134	11,3
	45-49	172	14,5
	50-54	168	14,2
	55-59	162	13,7
	60-64	91	7,7
	65-69	49	4,1
	70-74	31	2,6
	75+	17	1,4
Sex/gender			
	Male	613	51,8
	Female	570	48,2
Area of residency			
	Large city	411	34,7
	Small city	331	28,0
	Densely populated area	243	20,5
	In the country (rural area)	198	16,7
Residency (proximity to aquaculture)			
	Residing in an aquaculture municipality	565	47.8
	Residing in a non-aquaculture municipality	618	52.2
County			
	Viken	69	5,8
	Oslo	61	5,2
	Innlandet	61	5,2
	Vestfold og Telemark	68	5,7
	Agder	75	6,3
	Rogaland	139	11,7
	Vestland	211	17,8
	Møre og Romsdal	73	6,2
	Trøndelag	140	11,8
	Nordland	84	7,1
	Troms og Finnmark	202	17,1
Education			
	Primary and lower secondary school (1-10)	42	3,6
	Upper secondary school (11-13)	317	26,8
	College/University (Bachelor's degree)	532	45,0
	College/University (Master's degree)	292	24,7

**Table 2:** Overview of respondent distribution in terms of age, gender, area of residency, and education for Tasmania data.

Variable		N	Percent
Age group (years)			
	18-20	5	1,2
	21-24	10	2,5
	25-29	21	5,2
	30-34	41	10,1
	35-39	60	14,8
	40-44	48	11,8
	45-49	43	10,6
	50-54	41	10,1
	55-59	31	7,6
	60-64	36	8,9
	65-69	31	7,6
	70-74	23	5,7
	75+	16	3,9
Sex/gender			
	Male	170	41,9
	Female	234	57,6
	Non-binary	2	0,5
Area of residency			
	Tasmania South	212	52,2
	Tasmania North	129	31,8
	Tasmania North West	65	16,0
Education			
	Less than 10 years	10	2,5
	Completed 10 or equivalent	45	11,1
	Completed 12 year or equivalent	62	15,3
	TAFE/Trade qualification	121	29,8
	University Degree or higher	168	41,4

**Table 3:** Overview of respondent distribution in terms of age, gender, area of residency, and education for Iceland data.

Variable		N	Percent
Age group (years)			
	18-20	6	1,2
	21-24	20	4,0
	25-29	18	3,6
	30-34	39	7,9
	35-39	29	7,7
	40-44	33	6,7
	45-49	39	7,9
	50-54	50	10,1
	55-59	46	9,3
	60-64	52	10,5
	65-69	69	13,9
	70-74	46	9,3
	75+	40	8,1
Sex/gender			
	Male	221	44,6
	Female	275	55,4
Area of residency			
	Reykjavik and surroundings	344	69,4
	Other/rural	152	30,6
Education			
	Primary school	31	7,0
	Secondary school	212	48,0
	College/University	199	45,0

For a comprehensive list of all variables included in the data and their corresponding countries, see the codebook file.

## 4 Selected Results

**Fig. 1:**
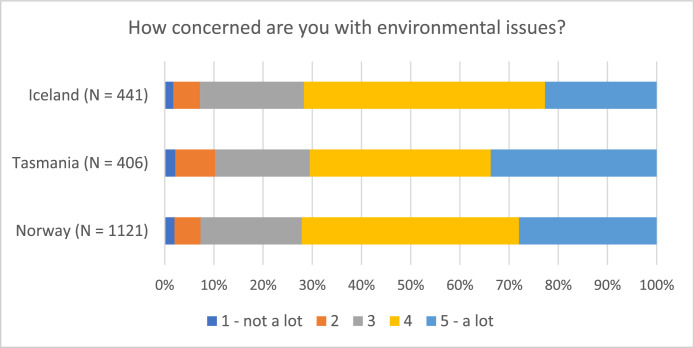
Respondents’ self-reported concern with environmental issues (in general) (“On a scale from 1 to 5, how concerned are you with environmental issues?”).

**Fig. 2:**
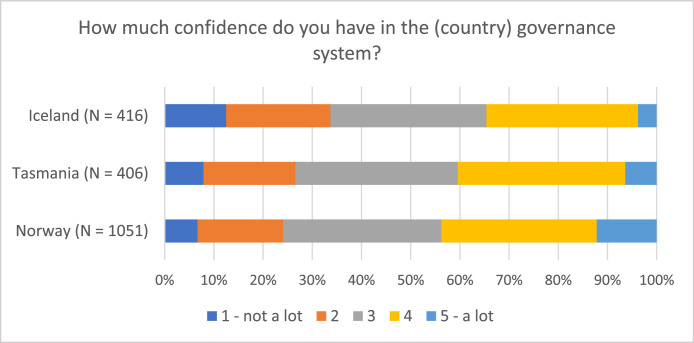
Respondents’ self-reported confidence in governance system (“On a scale from 1 to 5, how much confidence do you have in the Norwegian/Tasmanian/Icelandic governance system?”).

**Fig. 3:**
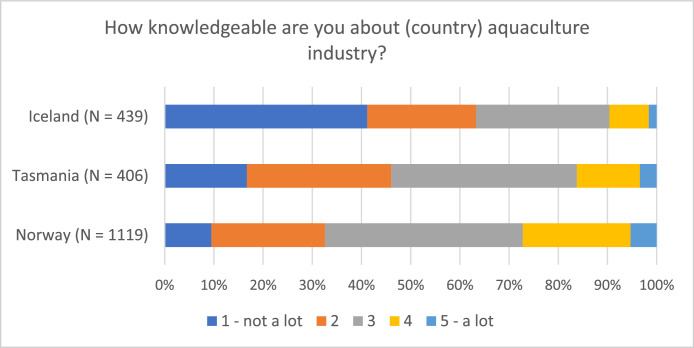
Respondents’ self-reported knowledge about the salmon aquaculture industry (“On a scale from 1 to 5, how knowledgeable are you about the Norwegian/Tasmanian/Icelandic salmon aquaculture industry?”).

The survey questions gauging attitudes towards the salmon aquaculture industry and its production were standardized across the three countries, albeit presented in different languages. Despite variations in the sizes of respondent groups, the findings are conducive to comparison on key subjects, including inquiries about respondents’ self-reported trust in the governance system of each country, environmental concerns, and their knowledge of the industry (Fig. 1, 2, and 3).

**Fig. 4:**
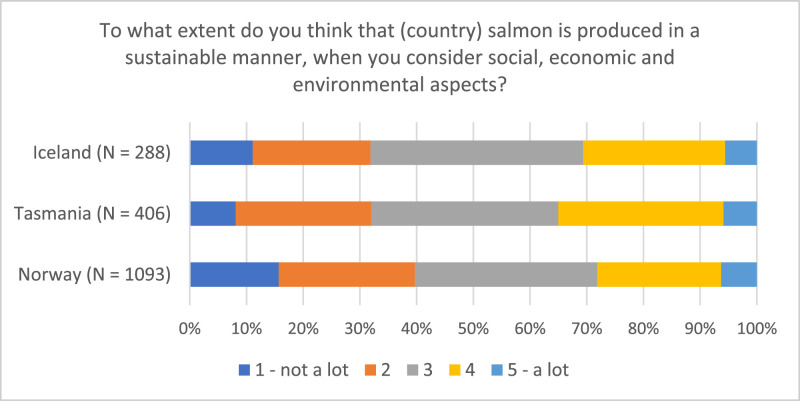
Respondents’ opinion on salmon being produced in a sustainable manner (“On a scale from 1 to 5, to what extent do you think that Norwegian/Tasmanian/Icelandic salmon is produced in a sustainable manner, when you consider social, economic, and environmental aspects?”).

**Fig. 5:**
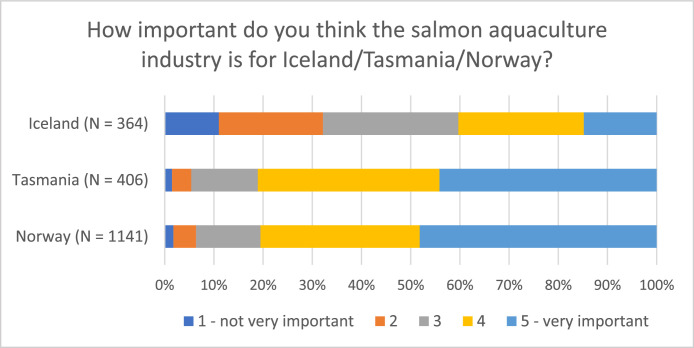
Respondent's opinion of industry importance (on a national level) (“On a scale from 1 to 5, how important do you think the salmon aquaculture industry is for Norway/Tasmania/Iceland?”).

**Fig. 6:**
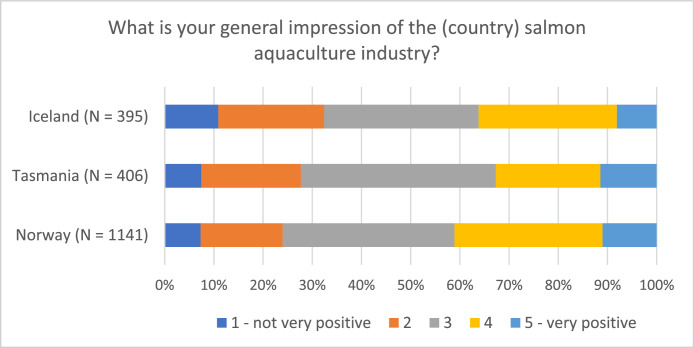
Respondents’ general impression of the salmon aquaculture industry (“On a scale from 1 to 5, what is your general impression of the Norwegian/Tasmanian/Icelandic salmon aquaculture industry?”).

**Fig. 7:**
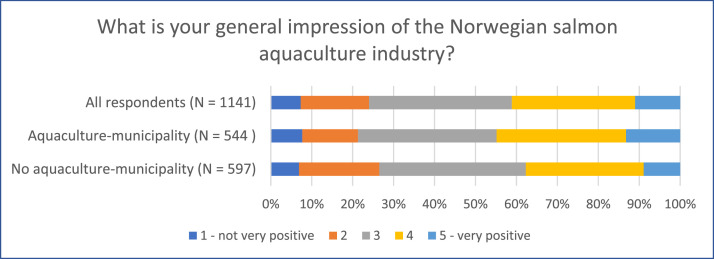
General impression of the aquaculture industry, distribution among all Norwegian respondents, and the division between respondents living in municipalities with and without aquaculture production.

Examining the distribution of respondents on various topics provides valuable insights into both similarities and differences among the countries at a national level (see Fig. 4, Fig. 5, Fig. 6). Despite the largely uniform production process of farmed salmon across sites (for example Norway, Iceland, and Tasmania), attitudes and perceptions of the industry can vary due to contextual differences. The data further reveals variations within specific respondent groups, illustrated by the proximity to the industry within the Norwegian dataset (see Fig. 7)

## 5 Experimental Design, Materials and Methods

The surveys were conducted between April and September 2020 in Norway, Tasmania, and Iceland. A Norwegian survey company (Norfakta) administered the survey in collaboration with survey companies in Tasmania (Myriad Research) and Iceland (Gallup Iceland). Local survey companies were used to secure a sufficient number of respondents in each country. The survey was an online survey, where respondents received an email with a link to the survey, which was provided in the native language. Recruitment of respondents was carried out by the survey companies using their own algorithms and programs, although it sought a representative selection of respondents in terms of gender and age distribution, and for Iceland and Tasmania: also, in terms of geographical distribution. The data provided by the survey companies included only completed survey responses; however, the total response rate is unknown. Some of the differences in respondents’ distribution, specifically for the Norwegian data, is due to the requirements given to the survey company.

The minimum number of respondents from each country and each county (Norway) were decided by the research group. For Norway, a minimum of 800 respondents were required, with at least 60 respondents from each county. However, a higher number was needed from the counties of “Hordaland” and “Troms” to conduct a separate comparative analysis at the local/regional level. For both Tasmania and Iceland, a minimum of 400 respondents was required to ensure statistical significance of the population sample.

The geographical distribution of the Norwegian respondents is presented in Table 1 (county) and ranged from 61 to 211 per county. Two factors account for the variations among counties: Firstly, in 2020, several counties were merged, impacting the distribution. Secondly, additional questions (Q21-28) pertaining to the aquaculture industry in respondents’ local communities were only given to respondents residing in the two specific counties in the eastern (“Hordaland”) and northern (“Troms”) parts of Norway. Consequently, a higher response rate was necessary for these groups. The analysis of these additional questions is not included in Olsen et al. [1] but will be presented in a separate publication.

Regarding the Norwegian data, we computed a variable to differentiate respondents residing near aquaculture production sites from those living elsewhere. This variable operates on the municipal level and is derived from respondents’ reported postcodes (which we recoded into municipality) and geographical data on aquaculture production. This variable aligns with the micro-level focus of the social license literature, which primarily examines the relationship between a company and the local community [1,3]. Its purpose was to investigate differences in perceptions at the local level.

The survey questions were based on topics and elements derived from social license literature (not limited to, but with notable contributions from [3], [4], [5], [6], [7], [8], [9], [10], [11], [12]), and the project team's extensive research experience (and previous work) on topics related to the aquaculture industry, social acceptance, and legitimacy. Survey questions and design were also adapted to suit the context of aquaculture in the regions data were collected for.

Survey questions concerning attitudes towards the aquaculture industry were mainly asked using a five-point Likert scale (all moving from “1” negative to “5” positive) with the additional option of answering “I don't know”/”I don't want to answer”. The ends of each scale were labeled (“1 - Not a lot”, “5 - A lot”) but “2”, “3” and “4” had no labels. See survey codebook for all labels (English version) and additional response options [13].

## Limitations

There were some variations in the available response options for “I don't know/I don't want to answer/uncertain” for the three regions. Specifically, Norwegian respondents had the option of selecting “I don't know”, while Icelandic participants could choose between “I don't know” and “I don't want to answer”, or even opt to provide no response. However, respondents from Tasmania were not offered the “I don't know” option for all questions. During the analysis process, all instances of “I don't know” options are treated as missing data.

There are variations in both the types and extent of demographic information gathered from the respondents from the three survey companies. Consequently, conducting analyses on respondent attribute variables, such as level of education and income, poses challenges when comparing across the countries.

The intentional uneven geographical distribution of respondents across the three countries (especially for the Norwegian data) can be viewed as a limitation. The reason for the skewed distribution was to allow for an additional in-depth comparative study of two Norwegian counties.

## Ethics Statement

This study was approved by SIKT (former NSD) – Norwegian Agency for Shared Services in Education and Research (ethics approvement for research), reference nr. 405082. Informed consent was obtained from respondents by the survey companies distributing the surveys in Norway, Iceland, and Tasmania.

## CRediT authorship contribution statement

**Marit Schei Olsen:** Methodology, Writing – original draft, Writing – review & editing, Data curation, Visualization. **Eirik Mikkelsen:** Methodology, Writing – review & editing. **Karen A. Alexander:** Methodology, Writing – review & editing. **Ragnheidur Thorarinsdottir:** Methodology, Writing – review & editing. **Tonje C. Osmundsen:** Methodology, Writing – review & editing, Project administration.

## Data Availability

Survey data on attitudes towards salmon aquaculture industry in Norway, Iceland, and Tasmania (AU) (Original data) (Zenodo)

## Appendix Supplementary materials

Image, application 1

Image, application 2

## Footnotes

1 For the Norwegian respondents there are additional individual characteristics on household size, the number of children in household, and how often they consumed salmon. For the Icelandic respondents, additional characteristics include household size.
