# Supplementary material for: Survey data on public perceptions of salmon aquaculture industry in Norway, Tasmania, and Iceland
Source: Data Brief. 2024 Jan 15;53:110067. doi: 10.1016/j.dib.2024.110067 (PMC10838686; doi:10.1016/j.dib.2024.110067)
Supplement: Supplementary file 1 [file mmc1.docx]

# **Codebook – Survey data**

**Key information**

*Variable name*

Variable description

Item value description

# Respondent information, demographic variables, and weight variables:

Listed below are the variables related to respondents’ demographic information and additional variables for information about respondents. Some of these were provided by the survey company (existing panel data), some were given to respondents to answer, and some have been computed/recoded by the research group. Some of these variables are only valid for respondents from one or more countries, which is commented on in variable and item value description.

**Repondent ID**

*CaseNR*

Case number, random unique identification number for each respondents. The codes also reflects the respondents country of residence (0-1183 = Norway, first digit 2xxx (2001-2406) = Tasmania, first digit 3xxx (3001-3496) = Iceland).

**Respondents’ country of residence**

*Country*

Respondents’ country, computed variable.

1 = Norway

2 = Tasmania/Australia

3 = Iceland

**County of residency (county divisions before 2020)** (Norway)

*County*

Respondents’ county, divisions before 2020. Provided by survey company. (Norway)

1 = Østfold

2 = Akershus

3 = Oslo

4 = Hedmark

5 = Oppland

6 = Buskerud

7 = Vestfold

8 = Telemark

9 = Aust-Agder

10 = Vest-Agder

11 = Rogaland

12 = Hordaland

13 = (not used – left blanc)

14 = Sogn og Fjordane

15 = Møre og Romsdal

16 = Sør-Trøndelag

17 = Nord-Trøndelag

18 = Nordland

19 = Troms

20 = Finnmark

**County of residency (county divisions after 2020)** (Norway)

*County_new*

Respondents’ county, divisions after 2020. Provided by survey company. (Norway)

1 = Viken (Østfold, Akershus, Buskerud)

2 = Oslo

3 = Innlandet (Hedmark, Oppland)

4 = Vestfold og Telemark

5 = Agder (Vest-Agder, Aust-Agder)

6 = Rogaland

7 = Vestland (Hordaland, Sogn og Fjordane)

8 = Møre og Romsdal

9 = Trøndelag

10 = Nordland

11 = Troms og Finnmark

**Aquaculture municipality** (Norway) (computed variable)

*AquaMunicipality*

Computed variable for dividing Norwegian respondents living in aquaculture municipality or non-aquaculture municipality. Based on reported post codes (not included in raw data) and the Aquaculture Fund distribution from 2020 reporting on municipalities with aquaculture production.

0 = Non-aquaculture municipality

1 = Aquaculture municipality

**Household income: What would you estimate your household’s total broken income at per year? (total income before taxes and deductions)** (Norway)

*Income*

Household income for respondents from Norway, provided in NOK.

1 = Up to 300 000 NOK

2 = 300 000 – 499 999 NOK

3 = 500 000 – 799 999 NOK

4 = 800 000 – 999 999 NOK

5 = 1 000 000 – 1 499 999 NOK

6 = 1 500 000 NOK or more

7 = Do not want to state income

8 = I don’t know

**Household income** (Iceland)

*FamIncome*

Household income for respondents from Iceland, provided in ISK.

1 = Less than 400 000 ISK

2 = 400 000 – 549 999 ISK

3 = 550 000 – 799 999 ISK

4 = 800 000 – 999 999 ISK

5 = 1 000 000 – 1 249 999 ISK

6 = 1 250 000 – 1 499 999 ISK

7 = 1 500 000 ISK or more

98 = I don’t want to state income

99 = I don’t know

**How many people in your household?** (Norway)

*PeopleHousehold*

The number of people in respondents’ household (Norway).

1 = 1 person

2 = 2 persons

3 = 3 persons

4 = 4 persons

5 = 5 persons

6 = 6 persons

7 = 7 persons

8 = 8 persons

9 = 9 people or more

**How many children under the age of 18 living at home are in the household?** (Norway)

*Children*

The number of children in respondents’ household (Norway).

1 = No children

2 = 1 child

3 = 2 children

4 = 3 children

5 = 4 children

6 = 5 children

7 = 6 children or more

**How often do you eat salmon? Please include for breakfast, lunch, and dinner, at restaurants as well as at home (all meals)**

*SalmonConsume*

Indicates how often respondents eat salmon (all meals). (Norway)

1. = Three times a week or more
2. = Twice a week
3. = Once a week
4. = 2-3 times a month
5. = Once a month
6. = Every other month
7. = Every third month
8. = Less often
9. = Never eats

**What is your highest education?** (Norway)

*Education_Norway*

Level of education for respondents from Norway.

1 = Primary and lower secondary school (1-10)

2 = Upper secondary school (11-13)

3 = College/University (Bachelor)

4 = College/University (Master or higher)

**What is the highest level of education you have obtained (or currently studying towards)?** (Tasmania)

*Education_Tasmania*

Level of education for respondents from Tasmania.

1 = Less than year 10

2 = Completed year 10 or equivalent

3 = Completed year 12 or equivalent

4 = TAFE/Trade qualification

5 = University degree or higher

6 = Other

**What is your highest education?** (Iceland)

*Education_Iceland*

Level of education for respondents from Iceland.

1. = Primary school
2. = Secondary School/High School
3. = College/University
4. = I don’t want to answer
5. = I don’t know

**Where do you live?** (Norway)

*AreaResidency*

Reflects if respondents live in an area which is urban/rural. (Norway)

1 = Large city

2 = Small city

3 = Densely populated area

4 = In the country/rural area

**Which best describes where you live?** (Tasmania)

*Residence_Tasmania*

Indicate in which area the respondents from Tasmania resides. Originally provided by a string variable, which has been recoded into a numeric variable.

1 = Tasmania south

2 = Tasmania North

3 = Tasmania North West

**Area of residency** (Iceland) (recoded variable)

*Residence_Iceland*

Indicate in which area the respondents from Iceland resides. Originally provided by postcodes, which has been recoded into urban (Reykjavik and surrounding area – postal codes 101-225 + 270-276) and more rural (other) areas.

0 = Rural areas/other

1 = Reykjavik and surroundings

**Are you…?** (sex/gender)

*Sex*

Respondents gender.

1 = Male

2 = Female

3 = Non-binary (option provided only for respondents from Tasmania)

**What is your age?** (open-ended for Norway and Iceland, group options for Tasmania. Recoded into groups for all three countries)

*Age_group*

Age categories in groups.

1 = 18-20

2 = 21-24

3 = 25-29

4 = 30-34

5 = 35-39

6 = 40-44

7 = 45-49

8 = 50-54

9 = 55-59

10 = 60-64

11 = 65-69

12 = 70-75

13 = 75+

**Weight variable for Norwegian data**

*Sysweight.1*

Norfakta provided this variable for the purpose of weighting data collected from Norwegian respondents. The variable is calculated based on geographical distribution (county – division prior to 2020), gender, and age (data from Statistics Norway). Please note that the raw data has not undergone any weighting adjustments.

**Weight variable for Icelandic data**

*vigt*

Gallup Island provided this variable for the purpose of weighting data collected from Icelandic respondents. The variable is calculated based on age, gender, education, and residency (data from Statistics Iceland). Please note that the raw data has not undergone any weighting adjustments.

# Respondents’ confidence in governance system, concern for environmental issues, and knowledge of aquaculture industry

(Questions provided to all respondents)

**Confidence in governance system:** **On a scale from 1 to 5, how much confidence do you have in the (country) governance system?**

*ConfidenceGov*

Level of confidence in country governance system.

1 = 1 Not a lot

2 = 2

3 = 3

4 = 4

5 = 5 A lot

6 = I don’t know/insecure

98 = I don’t want to answer

99 = I don’t know

**Concerned with environmental issues: On a scale from 1 to 5, how concerned are you with environmental issues?**

*EnvironmentConc*

Level of concern for environmental issues (in general).

1 = 1 Not a lot

2 = 2

3 = 3

4 = 4

5 = 5 A lot

6 = I don’t know/insecure

98 = I don’t want to answer

99 = I don’t know

**Knowledge of aquaculture industry: On a scale from 1 to 5, how knowledgeable are you about (country) aquaculture industry?**

*KnowInd*

Level of knowledge of salmon aquaculture industry.

1 = 1 Not a lot

2 = 2

3 = 3

4 = 4

5 = 5 A lot

6 = I don’t know/insecure

98 = I don’t want to answer

99 = I don’t know

# Survey variables for perceptions of salmon aquaculture industry in Norway/Tasmania/Iceland:

(Questions provided to all respondents)

**Q1. On a scale from 1 to 5, how easy is it to find information about the salmon aquaculture industry?**

*var1*

Indicate how easily respondents think information about industry is available.

1 = 1 Not very easy

2 = 2

3 = 3

4 = 4

5 = 5 Very easy

6 = I don’t know/insecure (Norway)

98 = I don’t want to answer (Iceland)

99 = I don’t know (Iceland)

**Q2. Where do you get your information about the aquaculture industry from? (select up to 3 responses from below)**

*var2_TV*

Indicate that TV is one of their information sources

0 /NA = No

1 = Yes/TV

*var2_Radio*

Indicate that Radio is one of their information sources

0/NA = No

1 = Yes/Radio

*var2_Newspaper*

Indicate that newspaper/magazines are one of their information sources

0/NA = No

1 = Yes/Newspaper

*var2_Internet*

Indicate that news sites on internet is one of their information sources

0/NA = No

1 = Yes/Internet news sites

*var2_SocMed*

Indicate that social media/internet is one of their information sources

0/NA = No

1 = Yes/Internet social media

*var2_Company*

Indicate that company websites/information is one of their information sources

0/NA = No

1 = Yes/Internet company websites

*var2_FamFriends*

Indicate that family /friends /colleagues is one of their information sources

0/NA = No

1 = Yes/Family, friends, colleagues

*var2_Other*

Indicate that other sources (than listed above) is one of their information sources. (comments are not included in raw data file)

0/NA = No

1 = Yes/Other

*var2_DontKnow*

Indicate that they do not know which information sources they could use to find information about the industry

0/NA = No

1 = Yes/I don’t know

*var2_None*

Indicate that none of the mentioned information sources are used/preferred (Option presented only for respondents from Iceland)

0/NA = No

1 = Yes/None of these – I don’t know

*var2_DontAnswer*

Indicate that respondents do not want to state which information sources are used/preferred (Option presented only for respondents from Iceland)

0/NA = No

1 = Yes/I don’t want to answer

**Q3. On a scale from 1 (not at all) to 5 (a lot), to what extent do you perceive the (country) salmon aquaculture industry as.. transparent?**

*var3*

Indicate to what extent the industry is perceived as transparent.

1 = 1 Not a lot

2 = 2

3 = 3

4 = 4

5 = 5 A lot

6 = I don’t know/insecure (Norway)

98 = I don’t want to answer (Iceland)

99 = I don’t know (Iceland)

**Q4. On a scale from 1 (not at all) to 5 (a lot), to what extent do you perceive the (country) salmon aquaculture industry as.. trustworthy?**

*var4*

Indicate to what extent the industry is perceived as trustworthy.

1 = 1 Not a lot

2 = 2

3 = 3

4 = 4

5 = 5 A lot

6 = I don’t know/insecure (Norway)

98 = I don’t want to answer (Iceland)

99 = I don’t know (Iceland)

**Q5. On a scale from 1 (not very positive) to 5 (very positive), what is your general impression of the (country) salmon aquaculture industry?**

*var5*

Indicate to what extent there is a positive general impression of the industry.

1 = 1 Not very positive

2 = 2

3 = 3

4 = 4

5 = 5 Very positive

6 = I don’t know/insecure (Norway)

98 = I don’t want to answer (Iceland)

99 = I don’t know (Iceland)

**Q6. On a scale from 1 (not very important) to 5 (very important), how important do you think the salmon aquaculture industry is for (country)?**

*var6*

Indicate to what extent there is an impression of the industry as important for Norway/Tasmania/Iceland.

1 = 1 Not very important

2 = 2

3 = 3

4 = 4

5 = 5 Very important

6 = I don’t know/insecure (Norway)

98 = I don’t want to answer (Iceland)

99 = I don’t know (Iceland)

**Q7. Which of these elements do you think the industry contributes the most with? (Please select as many as you think apply**)

*var7_Empl*

Indicate that ‘employment’ is one of the elements the industry contributes the most with.

0/NA = No

1 = Yes/Employment

*var7_RobComm*

Indicate that ‘robust communities’ is one of the elements the industry contributes the most with.

0/NA = No

1 = Yes/Robust communities

*var7_MunRevenues*

Indicate that ‘municipal revenues’ is one of the elements the industry contributes the most with.

0/NA = No

1 = Yes/Municipal revenues

*var7_StateRevenues*

Indicate that ‘state revenues’ is one of the elements the industry contributes the most with.

0/NA = No

1 = Yes/State revenues

*var7_BusinessAct*

Indicate that ‘increased business activity’ is one of the elements the industry contributes the most with.

0/NA = No

1 = Yes/Increased business activity

*var7_TechInnov*

Indicate that ‘technology development and innovation’ is one of the elements the industry contributes the most with.

0/NA = No

1 = Yes/Technology development and innovation

*var7_FoodProd*

Indicate that ‘food production’ is one of the elements the industry contributes the most with.

0/NA = No

1 = Yes/Food production

*var7_Other*

Indicate that there are other elements (than listed above) that the industry contributes the most with. (comments are not included in raw data file)

0/NA = No

1 = Yes/Other elements

*var7_None*

Indicate that they perceive the industry to contribute with none of these, or contributions to have little value (for Norwegian respondents)

0/NA = No

1 = Yes/None of these or little value

*var7_NoneLittleValue*

Indicate that they perceive the industry to contribute with none of these, or contributions to have little value (for Tasmanian respondents)

0/NA = No

1 = Yes/None of these or little value

*var7_DontAnswer*

Indicate that respondents did not want to answer. (for Icelandic respondents)

0/NA = No

1 = Yes/I don’t want to answer

*var7_DontKnow*

Indicate that respondents did not know what the industry contributes with the most. (for Icelandic respondents)

0/NA = No

1 = Yes/I don’t know

**Q8. All in all, on a scale from 1 (not very fairly) to 5 (very fairly), to what extent do you find that the economic benefits from (country) salmon aquaculture industry are distributed fairly, on a council level?**

*var8*

Indicate to what extent economic benefits from the industry is distributed fairly on a council level.

1 = 1 Not very fairly

2 = 2

3 = 3

4 = 4

5 = 5 Very fairly

6 = I don’t know/insecure (Norway)

98 = I don’t want to answer (Iceland)

99 = I don’t know (Iceland)

**Q9. All in all, on a scale from 1 (not very fairly) to 5 (very fairly), to what extent do you find that the economic benefits from (country) salmon aquaculture industry are distributed fairly, on a state level?**

*var9*

Indicate to what extent economic benefits from the industry is distributed fairly on a state level.

1 = 1 Not very fairly

2 = 2

3 = 3

4 = 4

5 = 5 Very fairly

6 = I don’t know/insecure (Norway)

98 = I don’t want to answer (Iceland)

99 = I don’t know (Iceland)

**Q10. On a scale from 1 (not very sustainable) to 5 (very sustainable), to what extent do you perceive the (country) salmon aquaculture industry as environmentally sustainable?**

*var10*

Indicate to what extent the industry is perceived as environmentally sustainable.

1 = 1 Not very sustainable

2 = 2

3 = 3

4 = 4

5 = 5 Very sustainable

6 = I don’t know/insecure (Norway)

98 = I don’t want to answer (Iceland)

99 = I don’t know (Iceland)

(Q11 omitted from raw data – comment field)

**Q12. On a scale from 1 (not at all) to 5 (a lot), to what extent do you find that the industry is behaving in accordance with... The expectations of society?**

*var12*

Indicate to what extent the industry behavior is perceived to be in accordance with the expectations from society.

1 = 1 Not a lot

2 = 2

3 = 3

4 = 4

5 = 5 A lot

6 = I don’t know/insecure (Norway)

98 = I don’t want to answer (Iceland)

99 = I don’t know (Iceland)

**Q13. On a scale from 1 (not at all) to 5 (a lot), to what extent do you find that the industry is behaving in accordance with... The expectations of the authorities?**

*var13*

Indicate to what extent the industry behavior is perceived to be in accordance with the expectations from the authorities.

1 = 1 Not a lot

2 = 2

3 = 3

4 = 4

5 = 5 A lot

6 = I don’t know/insecure (Norway)

98 = I don’t want to answer (Iceland)

99 = I don’t know (Iceland)

**Q14. On a scale from 1 (not very confident) to 5 (very confident), to what extent do you have confidence in how the (country) authorities regulate the salmon aquaculture industry?**

*var14*

Indicate to what extent respondents have confidence in how the authorities regulate the industry.

1 = 1 Not very confident

2 = 2

3 = 3

4 = 4

5 = 5 Very confident

6 = I don’t know/insecure (Norway)

98 = I don’t want to answer (Iceland)

99 = I don’t know (Iceland)

**Q15. On a scale from 1 (not very sustainable) to 5 (very sustainable), to what extent do you think that (country) salmon is produced in a sustainable manner, when you consider social, economic and environmental aspects?**

*var15*

Indicate to what extent the salmon is perceived to be produced in a sustainable manner considering social, economic, and environmental aspects.

1 = 1 Not a lot

2 = 2

3 = 3

4 = 4

5 = 5 A lot

6 = I don’t know/insecure (Norway)

98 = I don’t want to answer (Iceland)

99 = I don’t know (Iceland)

**Q16. On a scale from 1 (not a lot) to 5 (a lot), do you tolerate salmon aquaculture production in (country)?**

*var16*

Indicate to what extent the salmon aquaculture production is tolerated.

1 = 1 Not a lot

2 = 2

3 = 3

4 = 4

5 = 5 A lot

6 = I don’t know/insecure (Norway)

98 = I don’t want to answer (Iceland)

99 = I don’t know (Iceland)

**Q17. On a scale from 1 (not a lot) to 5 (a lot), do you accept salmon aquaculture production in (country)?**

*var17*

Indicate to what extent the salmon aquaculture production is accepted.

1 = 1 Not a lot

2 = 2

3 = 3

4 = 4

5 = 5 A lot

6 = I don’t know/insecure (Norway)

98 = I don’t want to answer (Iceland)

99 = I don’t know (Iceland)

**Q18. On a scale from 1 (not a lot) to 5 (a lot), would you like to see more salmon aquaculture production in (country)?**

*var18*

Indicate to what extent it is desirable with more salmon aquaculture production in their country.

1 = 1 Not a lot

2 = 2

3 = 3

4 = 4

5 = 5 A lot

6 = I don’t know/insecure (Norway)

98 = I don’t want to answer (Iceland)

99 = I don’t know (Iceland)

**Q19. On a scale from 1 (not a lot) to 5 (a lot), are you proud of (country) salmon aquaculture production?**

*var19*

Indicate to what extent respondents are proud of the salmon aquaculture production in their country.

1 = 1 Not a lot

2 = 2

3 = 3

4 = 4

5 = 5 A lot

6 = I don’t know/insecure (Norway)

98 = I don’t want to answer (Iceland)

99 = I don’t know (Iceland)

**Q20. On a scale from 1 (not a lot) to 5 (a lot), to what extent do you find that the salmon aquaculture industry has a financial significance for your local community?**

*var20*

Indicate to what extent the salmon aquaculture industry is perceived to have financial significance for local community.

1 = 1 Not a lot

2 = 2

3 = 3

4 = 4

5 = 5 A lot

6 = I don’t know/insecure (Norway)

98 = I don’t want to answer (Iceland)

99 = I don’t know (Iceland)

# Additional survey variables regarding salmon aquaculture industry in local community and industry contact:

These questions were given to all respondents from Iceland and Tasmania, while in Norway, only respondents from the counties “Troms” and “Hordaland” were asked to answer these. Respondents from other counties are missing/NA.

**Q21. On a scale from 1 (not a lot) to 5 (a lot), to what extent do you find that the salmon aquaculture industry locally listens to and respects community opinions?**

*var21*

Indicate to what extent the salmon aquaculture industry locally is perceived to listen to and respect community opinions.

1 = 1 Not a lot

2 = 2

3 = 3

4 = 4

5 = 5 A lot

6 = I don’t know/insecure (Norway)

98 = I don’t want to answer (Iceland)

99 = I don’t know (Iceland)

**Q22. On a scale from 1 (not a lot) to 5 (a lot), to what extent do you find that the salmon aquaculture industry locally is willing to change their practices in response to community concerns?**

*var22*

Indicate to what extent the salmon aquaculture industry locally is perceived to be willing to change their practices in response to community concerns.

1 = 1 Not a lot

2 = 2

3 = 3

4 = 4

5 = 5 A lot

6 = I don’t know/insecure (Norway)

98 = I don’t want to answer (Iceland)

99 = I don’t know (Iceland)

**Q23. On a scale from 1 (not a lot) to 5 (a lot), to what extent do you find that the salmon aquaculture industry locally is available for dialogue with the local community?**

*var23*

Indicate to what extent the salmon aquaculture industry locally is perceived to be available for dialogue with the local community.

1 = 1 Not a lot

2 = 2

3 = 3

4 = 4

5 = 5 A lot

6 = I don’t know/insecure (Norway)

98 = I don’t want to answer (Iceland)

99 = I don’t know (Iceland)

**Q24. On a scale from 1 (not a lot) to 5 (a lot), to what extent do you find that the salmon aquaculture industry locally initiates meetings with the community/local stakeholders?**

*var24*

Indicate to what extent the salmon aquaculture industry locally is perceived to initiate meetings with the community/local stakeholders.

1 = 1 Not a lot

2 = 2

3 = 3

4 = 4

5 = 5 A lot

6 = I don’t know/insecure (Norway)

98 = I don’t want to answer (Iceland)

99 = I don’t know (Iceland)

**Q25. On a scale from 1 (not a lot) to 5 (a lot), how much contact do you have with people working in the salmon aquaculture industry, formally or socially?**

*var25*

Indicate to what extent respondents have contact with people working in the salmon aquaculture industry.

1 = 1 Not a lot

2 = 2

3 = 3

4 = 4

5 = 5 A lot

6 = I don’t know/insecure (Norway)

98 = I don’t want to answer (Iceland)

99 = I don’t know (Iceland)

**Q26. On a scale from 1 (not a lot) to 5 (a lot), to what extent do you find contact with people working in the salmon aquaculture industry as positive?**

*var26*

Indicate to what extent respondents perceive contact with people working in the salmon aquaculture industry as positive.

1 = 1 Not a lot

2 = 2

3 = 3

4 = 4

5 = 5 A lot

6 = I don’t know/insecure (Norway)

98 = I don’t want to answer (Iceland)

99 = I don’t know (Iceland)

**Q27. On a scale from 1 (not a lot) to 5 (a lot), to what extent do you find that you have access to information from the local salmon aquaculture industry?**

*var27*

Indicate to what extent the respondents perceive to have access to information from the local salmon aquaculture industry.

1 = 1 Not a lot

2 = 2

3 = 3

4 = 4

5 = 5 A lot

6 = I don’t know/insecure (Norway)

98 = I don’t want to answer (Iceland)

99 = I don’t know (Iceland)

**Q28. On a scale from 1 (not a lot) to 5 (a lot), to what extent do you find that the local salmon aquaculture industry contributes to developing the local community?**

*var28*

Indicate to what extent the local salmon aquaculture industry is perceived to contribute in developing the local community.

1 = 1 Not a lot

2 = 2

3 = 3

4 = 4

5 = 5 A lot

6 = I don’t know/insecure (Norway)

98 = I don’t want to answer (Iceland)

99 = I don’t know (Iceland)
