# Supplementary material for: Survey data on public perceptions of salmon aquaculture industry in Norway, Tasmania, and Iceland
Source: Data Brief. 2024 Jan 15;53:110067. doi: 10.1016/j.dib.2024.110067 (PMC10838686; doi:10.1016/j.dib.2024.110067)
Supplement: Supplementary file 2 [file mmc2.docx]

# Survey (summarized questions)

## Demographics:

**Are you…?** (sex/gender)

- Male
- Female
- Non-binary (option provided only for respondents in Tasmania)

**What is your age?** (open-ended for Norway and Iceland, group options for Tasmania)

- 18-20
- 21-24
- 25-29
- 30-34
- 35-39
- 40-44
- 45-49
- 50-54
- 55-59
- 60-64
- 65-69
- 70-75
- 75+

**Postcode** (Norway and Iceland – not included in raw data to secure full anonymity)

**Where do you live?** (Norway)

- Large city
- Small city
- Densely populated area
- In the country/rural area

**Which best describes where you live?** (Tasmania)

- Tasmania south
- Tasmania north
- Tasmania north-west
- Outside of Tasmania ( = close survey)

**What would you estimate your household’s total broken income at per year? (total income before taxes and deductions)** (Norway)

- Up to 300 000 NOK
- 300 000 – 499 999 NOK
- 500 000 – 799 999 NOK
- 800 000 – 999 999 NOK
- 1 000 000 – 1 499 999 NOK
- 1 500 000 NOK or more
- I don’t want to state income
- I don’t know

**Household income** (Iceland)

- Less than 400 000 ISK
- 400 000 – 549 999 ISK
- 550 000 – 799 999 ISK
- 800 000 – 999 999 ISK
- 1 000 000 – 1 249 999 ISK
- 1 250 000 – 1 499 999 ISK
- 1 500 000 ISK or more
- I don’t want to state income
- I don’t know

**What is your highest education?** (Norway)

- Primary and lower secondary school (1-10)
- Upper secondary school (11-13)
- College/University (Bachelor)
- College/University (Master or higher)

**What is the highest level of education you have obtained (or currently studying towards)?** (Tasmania)

- Less than year 10
- Completed year 10 or equivalent
- Completed year 12 or equivalent
- TAFE/Trade qualification
- University (specify) degree or higher
- Other (specify)

**What is your highest education?** (Iceland)

- Primary school diploma
- High school diploma
- University degree
- I don’t want to answer
- I don’t know

**How many people in your household?** (Norway)

- 1 person
- 2 persons
- 3 persons
- 4 persons
- 5 persons
- 6 persons
- 7 persons
- 8 persons
- 9 persons or more

**How many children under the age of 18 living at home are in the household?** (Norway)

- No children
- 1 child
- 2 children
- 3 children
- 4 children
- 5 children
- 6 children or more

**How often do you eat salmon? Please include for breakfast, lunch, and dinner, at restaurants as well as at home (all meals)?** (Norway)

- Three times a week or more
- Two times a week
- Once a week
- 2-3 times a month
- Once a month
- Every other month
- Every third month
- Less often
- Never eats

## Questions on confidence in governance system, concern for environmental issues, and knowledge of the industry:

**On a scale from 1 (not a lot) to 5 (a lot), how much confidence do you have in the (country) governance system?**

- 1 Not a lot
- 2
- 3
- 4
- 5 A lot
- I don’t know / I don’t want to answer (Norway and Iceland only)

**On a scale from 1 (not a lot) to 5 (a lot), how concerned are you with environmental issues?**

- 1 Not a lot
- 2
- 3
- 4
- 5 A lot
- I don’t know / I don’t want to answer (Norway and Iceland only)

**On a scale from 1 (not a lot) to 5 (a lot), how knowledgeable are you about (country) aquaculture industry?**

- 1 Not a lot
- 2
- 3
- 4
- 5 A lot
- I don’t know / I don’t want to answer (Norway and Iceland only)

## Questions about the salmon aquaculture industry:

**Q1. On a scale from 1 (not very easy) to 5 (very easy), how easy is it to find information about the salmon aquaculture industry?**

- 1 Not very easy
- 2
- 3
- 4
- 5 Very easy
- I don’t know / I don’t want to answer (Norway and Iceland only)

**Q2. Where do you get your information about the aquaculture industry from? (select up to 3 responses from below)**

- TV
- Radio
- Printed newspaper/magazines
- Internet – news sites
- Internet – social media
- Internet – company websites/information
- Family /friends/colleagues
- Other – (comments)
- None of these / I don’t know

**Q3. On a scale from 1 (not at all) to 5 (a lot), to what extent do you perceive the (country) salmon aquaculture industry as.. transparent?**

- 1 Not a lot
- 2
- 3
- 4
- 5 A lot
- I don’t know / I don’t want to answer (Norway and Iceland only)

**Q4. On a scale from 1 (not at all) to 5 (a lot), to what extent do you perceive the (country) salmon aquaculture industry as.. trustworthy?**

- 1 Not a lot
- 2
- 3
- 4
- 5 A lot
- I don’t know / I don’t want to answer (Norway and Iceland only)

**Q5. On a scale from 1 (not very positive) to 5 (very positive), what is your general impression of the (country) salmon aquaculture industry?**

- 1 Not very positive
- 2
- 3
- 4
- 5 Very positive
- I don’t know / I don’t want to answer (Norway and Iceland only)

**Q6. On a scale from 1 (not very important) to 5 (very important), how important do you think the salmon aquaculture industry is for (country)?**

- 1 Not very important
- 2
- 3
- 4
- 5 Very important
- I don’t know / I don’t want to answer (Norway and Iceland only)

**Q7. Which of these elements do you think the industry contributes the most with? (Please select as many as you think apply**)

- Employment
- Robust communities
- Municipal revenues
- State revenues
- Increased business activity
- Technology development and innovation
- Food production
- Other
- None of these / little value

**Q8. All in all, on a scale from 1 (not very fairly) to 5 (very fairly), to what extent do you find that the economic benefits from (country) salmon aquaculture industry are distributed fairly, on a council level?**

- 1 Not very fairly
- 2
- 3
- 4
- 5 Very fairly
- I don’t know / I don’t want to answer (Norway and Iceland only)

**Q9. All in all, on a scale from 1 (not very fairly) to 5 (very fairly), to what extent do you find that the economic benefits from (country) salmon aquaculture industry are distributed fairly, on a state level?**

- 1 Not very fairly
- 2
- 3
- 4
- 5 Very fairly
- I don’t know / I don’t want to answer (Norway and Iceland only)

**Q10. On a scale from 1 (not very sustainable) to 5 (very sustainable), to what extent do you perceive the (country) salmon aquaculture industry as environmentally sustainable?**

- 1 Not very sustainable
- 2
- 3
- 4
- 5 Very sustainable
- I don’t know / I don’t want to answer (Norway and Iceland only)

**Q11. In your opinion, in which areas should the industry become more sustainable? (comment field)** (Not included in raw data)

**Q12. On a scale from 1 (not at all) to 5 (a lot), to what extent do you find that the industry is behaving in accordance with... The expectations of society?**

- 1 Not at all
- 2
- 3
- 4
- 5 A lot
- I don’t know / I don’t want to answer (Norway and Iceland only)

**Q13. On a scale from 1 (not at all) to 5 (a lot), to what extent do you find that the industry is behaving in accordance with... The expectations of the authorities?**

- 1 Not at all
- 2
- 3
- 4
- 5 A lot
- I don’t know / I don’t want to answer (Norway and Iceland only)

**Q14. On a scale from 1 (not very confident) to 5 (very confident), to what extent do you have confidence in how the (country) authorities regulate the salmon aquaculture industry?**

- 1 Not very confident
- 2
- 3
- 4
- 5 Very confident
- I don’t know / I don’t want to answer (Norway and Iceland only)

**Q15. On a scale from 1 (not very sustainable) to 5 (very sustainable), to what extent do you think that (country) salmon is produced in a sustainable manner, when you consider social, economic and environmental aspects?**

- 1 Not very sustainable
- 2
- 3
- 4
- 5 Very sustainable
- I don’t know / I don’t want to answer (Norway and Iceland only)

**Q16. On a scale from 1 (not a lot) to 5 (a lot), do you tolerate salmon aquaculture production in (country)?**

- 1 Not a lot
- 2
- 3
- 4
- 5 A lot
- I don’t know / I don’t want to answer (Norway and Iceland only)

**Q17. On a scale from 1 (not a lot) to 5 (a lot), do you accept salmon aquaculture production in (country)?**

- 1 Not a lot
- 2
- 3
- 4
- 5 A lot
- I don’t know / I don’t want to answer (Norway and Iceland only)

**Q18. On a scale from 1 (not a lot) to 5 (a lot), would you like to see more salmon aquaculture production in (country)?**

- 1 Not a lot
- 2
- 3
- 4
- 5 A lot
- I don’t know / I don’t want to answer (Norway and Iceland only)

**Q19. On a scale from 1 (not a lot) to 5 (a lot), are you proud of (country) salmon aquaculture production?**

- 1 Not a lot
- 2
- 3
- 4
- 5 A lot
- I don’t know / I don’t want to answer (Norway and Iceland only)

**Q20. On a scale from 1 (not a lot) to 5 (a lot), to what extent do you find that the salmon aquaculture industry has a financial significance for your local community?**

- 1 Not a lot
- 2
- 3
- 4
- 5 A lot
- I don’t know / I don’t want to answer

## Additional questions regarding local community and industry contact:

These questions were given to all respondents from Iceland and Tasmania, while in Norway, only respondents from the counties “Troms” and “Hordaland” were asked to answer these.

**Q21. On a scale from 1 (not a lot) to 5 (a lot), to what extent do you find that the salmon aquaculture industry locally listens to and respects community opinions?**

- 1 Not a lot
- 2
- 3
- 4
- 5 A lot
- I don’t know / I don’t want to answer

**Q22. On a scale from 1 (not a lot) to 5 (a lot), to what extent do you find that the salmon aquaculture industry locally is willing to change their practices in response to community concerns?**

- 1 Not a lot
- 2
- 3
- 4
- 5 A lot
- I don’t know / I don’t want to answer

**Q23. On a scale from 1 (not a lot) to 5 (a lot), to what extent do you find that the salmon aquaculture industry locally is available for dialogue with the local community?**

- 1 Not a lot
- 2
- 3
- 4
- 5 A lot
- I don’t know / I don’t want to answer

**Q24. On a scale from 1 (not a lot) to 5 (a lot), to what extent do you find that the salmon aquaculture industry locally initiates meetings with the community/local stakeholders?**

- 1 Not a lot
- 2
- 3
- 4
- 5 A lot
- I don’t know / I don’t want to answer

**Q25. On a scale from 1 (not a lot) to 5 (a lot), how much contact do you have with people working in the salmon aquaculture industry, formally or socially?**

- 1 Not a lot
- 2
- 3
- 4
- 5 A lot
- I don’t know / I don’t want to answer

**Q26. On a scale from 1 (not a lot) to 5 (a lot), to what extent do you find contact with people working in the salmon aquaculture industry as positive?**

- 1 Not a lot
- 2
- 3
- 4
- 5 A lot
- I don’t know / I don’t want to answer

**Q27. On a scale from 1 (not a lot) to 5 (a lot), to what extent do you find that you have access to information from the local salmon aquaculture industry?**

- 1 Not a lot
- 2
- 3
- 4
- 5 A lot
- I don’t know / I don’t want to answer

**Q28. On a scale from 1 (not a lot) to 5 (a lot), to what extent do you find that the local salmon aquaculture industry contributes to developing the local community?**

- 1 Not a lot
- 2
- 3
- 4
- 5 A lot
- I don’t know / I don’t want to answer
